# Supplementary material for: In Silico Studies on Zinc Oxide Based Nanostructured Oil Carriers with Seed Extracts of Nigella sativa and Pimpinella anisum as Potential Inhibitors of 3CL Protease of SARS-CoV-2
Source: Molecules. 2022 Jul 4;27(13):4301. doi: 10.3390/molecules27134301 (PMC9268682; doi:10.3390/molecules27134301)
Supplement: Supplementary file 1 [file molecules-27-04301-s001.zip › molecules-1750771-supplementary.pdf]

Supplementary Materials

In Silico Studies on Zinc Oxide based Nanostructured Oil Carriers with Seed Extracts of *Nigella sativa* and *Pimpinella anisum* as Potential Inhibitors of 3CL Protease of SARS-CoV-2

Awatif A. Hendi [1], Promy Virk [2,*], Manal A. Awad [3], Mai Elobeid [2], Khalid M. O. Ortashi [4], Meznah M. Alanazi [1], Fatemah H. Alkallas [1], Maha Mohammad Almoneef [1] and Mohammed Aly Abdou [5]

[1] Department of Physics, College of Science, Princess Nourah Bint Abdulrahman University, P.O. Box 84428, Riyadh 11671, Saudi Arabia; aahindi@pnu.edu.sa (A.A.H.); mmalenazy@pnu.edu.sa (M.M.A.); fhalkallas@pnu.edu.sa (F.H.A.); mmalmoneef@pnu.edu.sa (M.M.A.)

[2] Department of Zoology, College of Sciences, King Saud University, P.O. Box: 2455, Riyadh,11451, Saudi Arabia; melobeid@ksu.edu.sa

[3] King Abdullah Institute for Nanotechnology, King Saud University, Riyadh 11451, Saudi Arabia; mawad@ksu.edu.sa

[4] Department of Chemical Engineering, King Saud University, Riyadh 11421, Saudi Arabia; ortashi9@ksu.edu.sa

[5] Department of Physics, College of Sciences, University of Bisha, P.O. Box 344, Bisha 61922, Saudi Arabia; m_abdou_eg@yahoo.com

* Correspondence: bvirk@ksu.edu.sa; Tel: +966-15-0220-5785

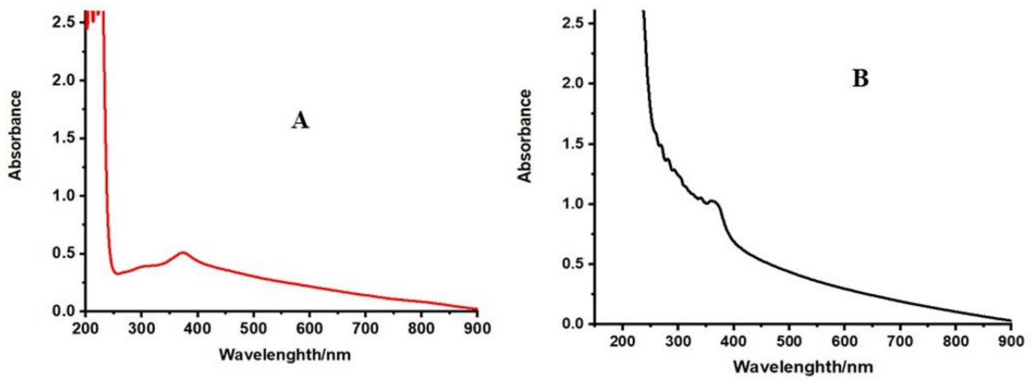

Figure S1. The UV–visible absorption spectra of synthesized (A) ZnO NPs (B) ZnO NPs–NLC.

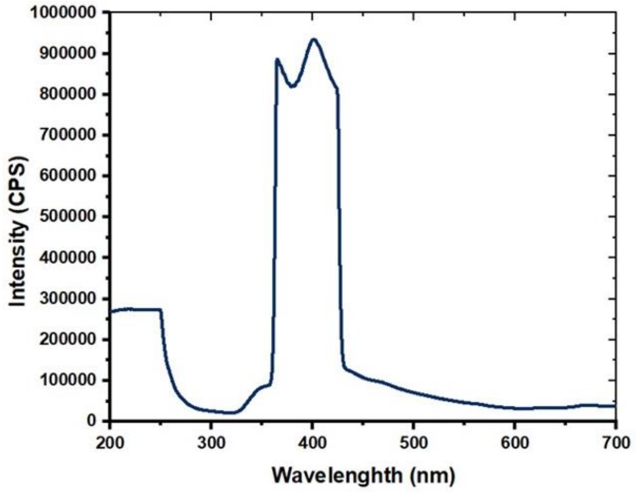

Figure S2. The emission spectrum of synthesized ZnO NPs.

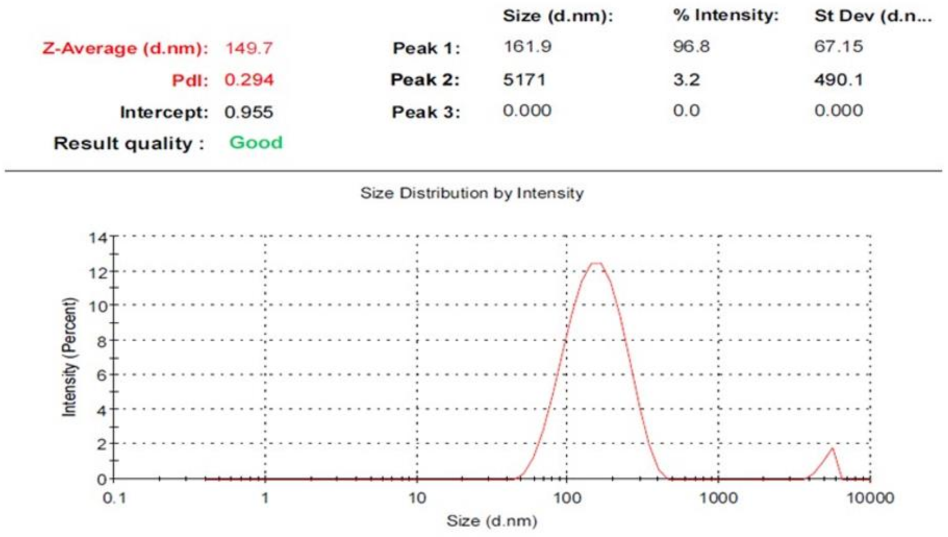

Figure S3. Particle size and polydispersity index of the ZnNPs.

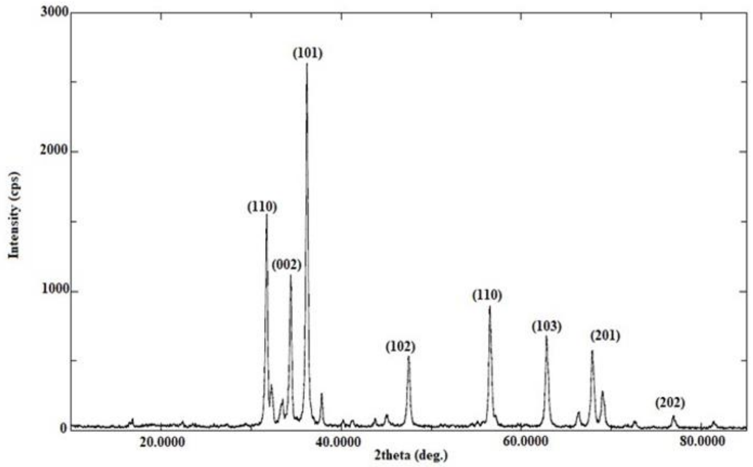

Figure S4. X-Ray diffraction pattern and diffraction angles peaks of synthesized ZnO NPs–NLC.

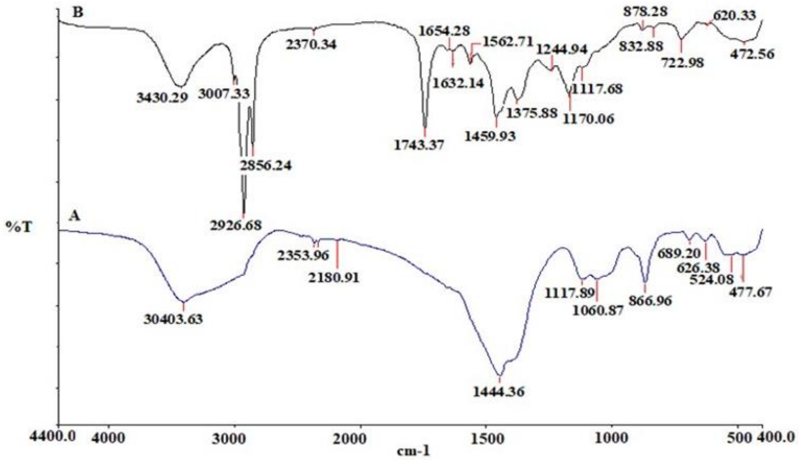

Figure S5. FT-IR spectra of synthesized (A) ZnO NPs (B) ZnO NPs–NLC.

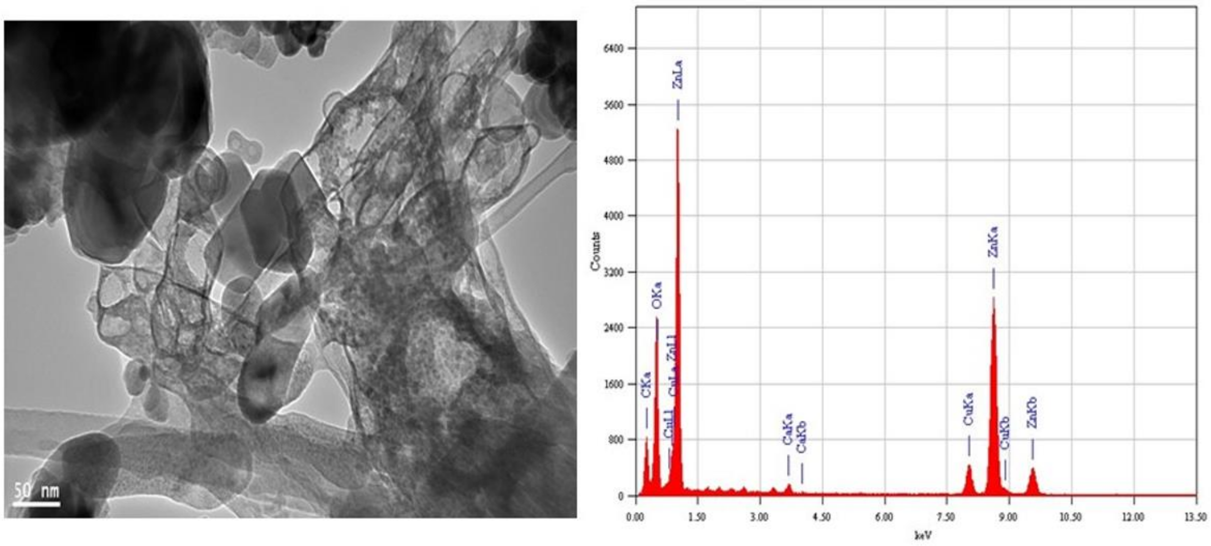

Figure S6. TEM micrograph and EDX spectrum of synthesized ZnO NPs.

Table S1: Structures of constituent compounds.

	**Bioactive compounds**
Pimpinella anisum	eugenol, *trans*-anethole, methylchavicol, anisaldehyde, estragole, coumarins, scopoletin, umbelliferone, estrols, terpene hydrocarbons, polyenes, and polyacetylenes
	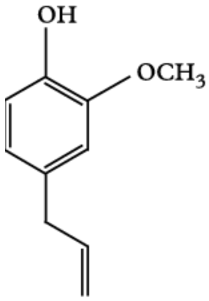<br>eugenol<br>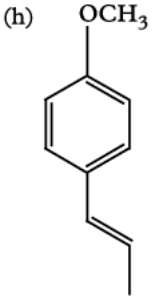<br>*trans*-anethole<br>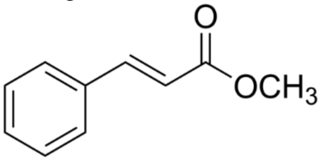<br>methylchavicol<br>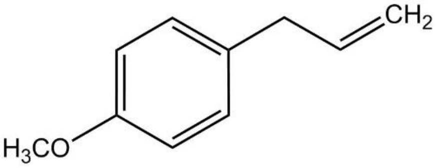<br>estragole<br>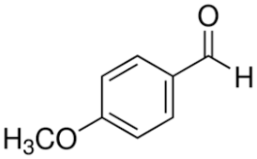<br>anisaldehyde<br>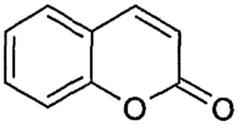<br>coumarin<br>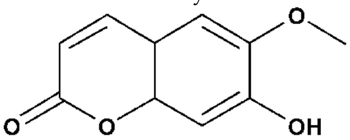<br>Scopoletin<br>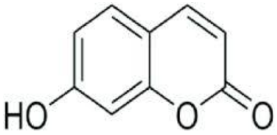<br>Umbelliferone<br>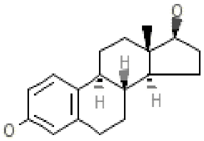<br>estrol<br>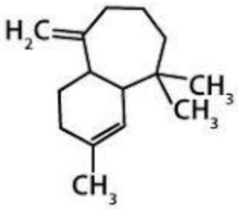<br>Sesquiterpene: Himachalene

Nigella Sativa	thymoquinone (30%-48%), thymohydroquinone, dithymoquinone, *p*-cymene (7%-15%), carvacrol (6%-12%), 4-terpineol (2%-7%), *t*-anethole (1%-4%), sesquiterpene longifolene (1%-8%) *α*-pinene and thymol
	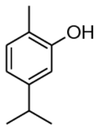 **Carvacrol** 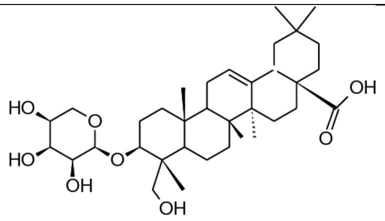 **δ–Hederin** 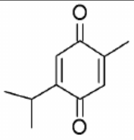 Thymoquinone 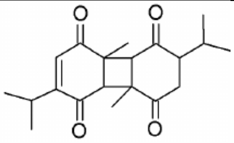 Dithymoquinone<br>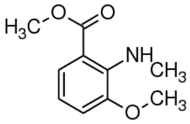 **Nigellone** 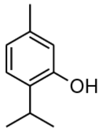 **Thymol** 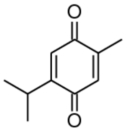 **Thymoquinone** 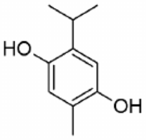 Thymohydroquinone 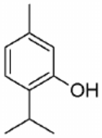 Thymol<br>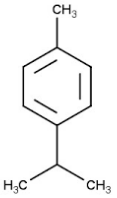 *p*-cymene 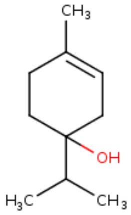 4-terpineol 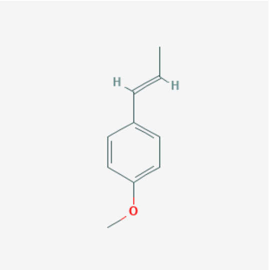 *t*-anethole<br>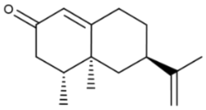<br>sesquiterpene longifolene
Zinc Oxide	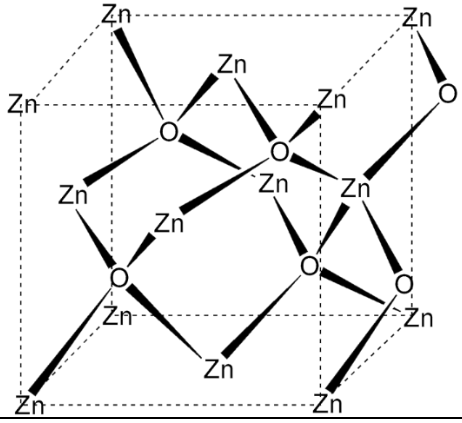

Olive Oil	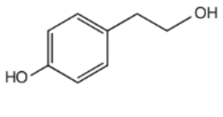 (Tyrosol) 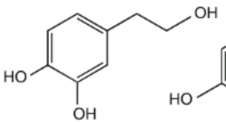 (Hydroxytyrosol) 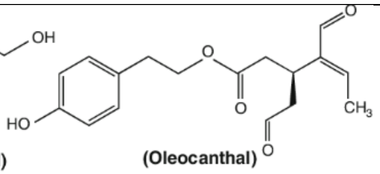 (Oleocanthal)<br>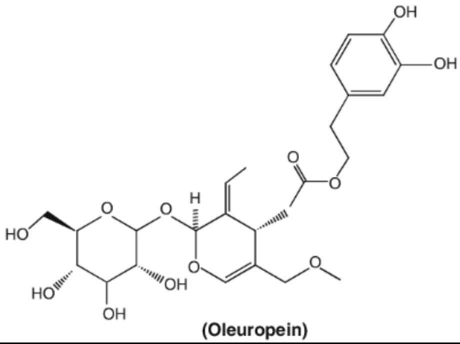 (Oleuropein)
